# What causes dyslexia? Identifying the causes and effective compensatory therapy

**DOI:** 10.3233/RNN-190939

**Published:** 2019-12-13

**Authors:** Reinhard Werth

**Affiliations:** Institute for Social Pediatrics and Adolescent Medicine Ludwig-Maximilians-University of Munich, Munich, Germany

**Keywords:** Dyslexia, causes, reading inability, therapy, children, eye movements, simultaneous recognition

## Abstract

**Background:**

Children are diagnosed as dyslexic when their reading performance is much below that which could be expected for their educational level and cannot be explained by a sensory, neurological or psychiatric deficit or by a low IQ. Although poor reading is a major obstacle to school and career achievement, the causes of dyslexia are unclear and traditional therapies are often unsuccessful. To determine the causes of dyslexia, experiments must demonstrate under which conditions a reading disorder occurs and whether the reading performance improves if these conditions are abolished or compensated. To avoid irreproducible results, experiments must be repeated and the effect size must be calculated.

**Objectives:**

The aims of the study were to investigate the rate and location of misread letters within pseudowords, prove the effectiveness of compensatory reading therapy and demonstrate the reproducibility of the experimental results. The influence of reading therapy on the rate of eye movements opposite to the reading direction was investigated and causes of a poor reading performance were identified.

**Methods:**

The rate and location of misread letters were investigated by tachystoscopic presentation of pseudowords containing between three and six letters. Presentation time, fixation time, and the time it takes to begin pronouncing the words (speech onset latency) were changed until 95% of the pseudowords were recognized correctly. On the basis of these results, the children learned a reading strategy that compensated the causes of the reading disorder. The therapy was demonstrated to be highly effective and it was shown that the results of the therapy were reproducible.

**Results:**

It was shown that misread letters occurred at all locations in pseudowords, regardless of the word's length. Inadequate fixation, excessively large saccadic amplitudes, reduced ability to simultaneously recognize a sequence of letters, a longer required fixation time and a longer required speech onset latency were all identified as causes of dyslexia. Each of the studies included in the meta-analysis were much more efficient than conventional therapeutic methods. The overall effect size with a value of Hedges' G = 1.72 showed that the therapy had a reproducible and stable effect.

**Conclusions:**

The causes of dyslexia can be revealed by a dual-intervention approach consisting of a pseudoword experiment and learning a compensatory reading strategy. Reading performance improves immediately if the identified causes of dyslexia are compensated by an appropriate reading therapy.

## 1 Introduction

According to conventional wisdom, the term *dyslexia* designates poor reading performance that cannot be explained by a primary visual deficit such as a visual field defect, reduced visual acuity, an eye movement disturbance, a hearing disorder, or a neurological or psychiatric disease. The reading performance is “substantially below that expected given the person’s chronological age, measured intelligence, and age-appropriate education” (Diagnostic and Statistical Manual of Mental Disorders, DSM 4, p. 50; Lyon et al. 2003; Fletcher 2009). This view has been revised in the Diagnostic and Statistical Manual DSM 5, where dyslexia is now regarded as a specific learning disorder that is indicated by “ ...   inaccurate and effortful word reading,  ...   difficulty understanding the meaning of what is read  ...  ” and “ ...   difficulty with spelling.” These difficulties must have persisted for at least six months and remain below the skills expected for the chronological age. The difficulties “ ...   are not better accounted for by intellectual disabilities, uncorrected visual or auditory acuity, other mental or neurological disorders, psychological adversity, lack in the proficiency in the language of academic instruction, or inadequate educational instruction  ...  ” (DSM 5 2013, p. 67). A link between the result of a reading test and the IQ is no longer required. According to these criteria, approximately 5% –15% of school children in the USA are dyslexic (Shaywitz et al., 1990; Katusic et al., 2001; Rutter et al., 2004). In Germany, the proportion of 4th graders with dyslexia is also estimated at 15% (Bos, 2012).

Questions arise on the causes underlying this kind of reading disorder. Reading is a complex skill that requires many different brain functions. The gaze must be directed approximately at the middle of the word or word segment that must be read so that as many letters of the word as possible are projected into the area of the retina, which has a sufficiently high visual acuity for reading. The field of attention has to be extended such that attention is directed to all letters that must be recognized. The word or word segment has to be fixated for a sufficiently long time such that the pattern and arrangement of the letters, their size, and their position within the word can be processed by the visual system. In addition, the shape and position of the letters of a word cannot be processed letter by letter. The pattern and position of a sequence of letters must be recognized almost simultaneously, which requires a separate brain capacity for simultaneous recognition (Poppelreuter, 1917; Werth, 2001, 2018). The simultaneous recognition of a sequence of letters is, however, a visual task that is made more difficult by lateral masking, also known as “crowding effect” (Stuart & Burian, 1962; Stromeyer & Julesz, 1972; Strasburger et al., 1991). The crowding effect reduces the ability to recognize a letter as the letter to be read is flanked on both sides by other letters.

Visually processed letter sequences must be associated with learned sound sequences and meanings stored in the memory. Finally, the words that have been read must be stored in memory and combined into sentences. A deficit in one or more of these abilities, required for reading, may cause a reading deficit. It is unclear whether abnormal eye movements are also a cause of dyslexia or whether abnormal eye movements are a consequence of other reading impairments (Pavlidis, 1981, 1985; Rainer, 1985; Eden et al., 1994; De Luca et al., 1999; Biscaldi et al., 1998, 2000; Hutzler et al., 2006; Blythe et al., 2018; Stein 2018a, b). It was also assumed that a deficit in shifting covert visual attention (Valdois et al., 2004; Buchholz & Davis, 2005), unusual foveal and parafoveal processing of visual stimuli, an enhanced visual crowding effect (Geiger & Lettwin, 1987; Atkinson, 1997; Lorusso et al., 2004), an impaired ability to process auditory stimuli (Tallal et al., 1993; Nagarajan et al., 1999; Tallal, 2000; King, et al., 2003), or a phonological impairment (Bruce, 1964; Wagner & Torgesen, 1987; Lundberg et al., 1988; Goswami & Bryant, 1990) play a causal role in the pathogenesis of dyslexia.

The experiments reported in this paper investigate (1) whether a reduced ability to recognize multiple letters simultaneously, (2) longer fixation times required to recognize multiple letters simultaneously, (3) longer time intervals required to establish the connection between letter sequences and sound sequences, and to retrieve them from memory (speech onset latency), and finally, (4) saccade amplitudes not adjusted to the ability to recognize a sequence of letters simultaneously may cause a reduced reading performance.

This paper is divided in three main sections. The first section describes a pseudoword experiment in 40 dyslexic children that examines the rate and position of misread letters in pseudowords consisting of 3 to 6 letters. It examines whether an increase in fixation time and/or a decrease in the number of letters along with an increase in the phoneme retrieval time, reduces the rate of letters read incorrectly. In the second section the effect of a new compensatory reading therapy is demonstrated. To this end, the children in the therapy group learn a reading strategy in which they divide the text into word segments that contain only as many letters as the children can recognize simultaneously. The children learn to fixate the word segments to be read for a sufficiently long time and start pronouncing the word segment only when they have reliably recognized it. It was investigated whether irregular eye movements (i. e. eye movements opposite to the reading direction) decreased when the reading capacity improved during therapy. However, if irregular eye movements do not decrease, despite an improvement of reading capacity, we cannot conclude that a poor reading skill is due to irregular eye movements. In the third section we adhered to the demands of the American Statistical Association and showed in a meta-analysis that the effect of the new compensatory reading therapy is repeatable and has the greatest effect size measured thus far.

In recent years, numerous objections have been raised against statistics that uses a *p*-value to decide whether a result should be regarded as significant or not (Nuzzo, 2014; Joannidis, 2005; Simmons et al., 2011; Wasserstein & Lazar, 2016). Therefore, the data were analyzed according to the requirements of the American Statistical Association (Wasserstein & Lazar, 2016). It strongly recommends replacing traditional statistics based on *p*-values and significance criteria with confidence intervals and effect sizes (Thompson, 1993; Lambdin, 2012). Therefore the effect sizes Cohen d and Hedges' g (Hedges & Olkin, 1985; Borenstein et al., 2009) were computed and the Hedges' - G summary effect was computed from three earlier studies and the present one. *P*-values are also reported, but they were not used to rejection or accept a hypothesis (Joannidis, 2005; Simmons et al., 2011; Nuzzo, 2014; Wasserstein & Lazar, 2016).

## 2 Experiment 1: The compensatory pseudoword experiment as a prerequisite for the compensatory reading therapy

The aim of the pseudoword experiment is to investigate which letters of the 3 to 6-letter pseudowords are misread, at which positions, and to detect the conditions under which poor readers are able to correctly read at least 95% of a list of pseudowords using the Celeco Software-Package for the Diagnosis and Therapy of Dyslexia (Werth et al., 2006, 2019).

### 2.1 Patients

Forty children (23 boys and 17 girls) aged between 8 and 15 years (mean age: 123. months; SD: 16 months) participated in the pseudoword experiment. All children were below the 16th percentile (1 SD) in the Zuerich Reading Test (ZLT). Only 2 children reached the 15th percentile). Fourteen were below the 6th percentile (1.5 SD), and 24 children were below the 2.5th percentile (i. e. 2 SD). All children were native German speakers and right-handed. They had no neurological, psychiatric, visual, or auditory deficits and no speech disorders. The children's IQs were within the normal range. The children were second-to-sixth graders who knew all individual letters, and were expected to read fluently but were far behind the required reading ability. The reading disabilities were not based on lack of teaching or inadequate educational instructions.

### 2.2 Methods

#### 2.2.1 Procedure

The compensatory pseudoword reading experiment investigated the rate and position of misread letters within pseudowords, and the conditions under which poor readers were able to correctly read at least 95% of a list of pseudowords using the Celeco Software-Package for the Diagnosis and Therapy of Dyslexia (Werth et al., 2006, 2019). Pronounceable 3-, 4-, 5-, and 6-letter pseudowords were presented at eye level. The distance between the eyes and the monitor was 40 cm. The words were black (luminance of 4 cd/m²; altitude 14 mm; space between types: 4 mm) on a background of 68 cd/m². Fixation of the word segments and saccadic eye movements were recorded using an infrared eye-tracking system (IRIS eye tracker; sampling rate: 500 Hz). Eye movements were monitored online, stored, and analyzed online and offline.

The presentation times of the pseudowords varied between 250 and 500 milliseconds. The sequences of the letters in the pseudowords also occurred in colloquial German words. Each trial began with the presentation of a fixation mark (luminance: 28 cd/m²) at the center of the monitor; the child was instructed to direct his/her gaze towards it. When the child maintained fixation, the fixation mark disappeared and was replaced by a pseudoword. The middle of the pseudoword was at the same location as the fixation mark. The children were to read the pseudoword aloud. If the child did not pronounce the word correctly, s/he was asked to spell the word and write it down. The children were instructed to not start pronouncing until they were sure of the word, and not start pronouncing immediately. After each pronunciation, they were asked to correct themselves, if necessary, within 5 to 10 seconds. To test the role of the recall time, a sound signal was given 3 seconds after the pseudoword appeared. The subjects were not supposed to start speaking until they heard the sound signal. After an interval between 5 to 10 seconds, the green fixation mark was presented again. When the child's gaze was on the fixation mark, a different pseudoword appeared for the same time interval as the previously shown pseudoword. In the first trial, a sequence of 20 pseudowords, consisting of 4 letters, was shown on the monitor. Each pseudoword was presented for 250 milliseconds. If 95% of the pseudowords were read correctly, a new sequence of 20, 5-letter pseudowords was presented for 250 milliseconds. If 95% of these pseudowords were read correctly, a different sequence of 20, 6-letter pseudowords was presented for 250 milliseconds. If only 80% to 90% of a sequence of pseudowords was read correctly, a different sequence of pseudowords of the same length was presented. The presentation time of each pseudoword in the new sequence was increased by 50 ms. If, for example, only 80% of a sequence of 4-letter pseudowords presented for 250 ms was read correctly, a different sequence of 4-letter pseudowords was shown. Each pseudoword was presented for 300 ms. If still less than 95% of this sequence of letters was read correctly, a new sequence of 4-letter pseudowords was presented. Each new pseudoword was presented for 350 ms. If less than 80% of a sequence of pseudowords was read correctly a different list of pseudowords was presented and the number of letters was reduced by one. Therefore, until 95% of a list of pseudowords was read correctly, the fixation times increased and/or the number of letters to be read was increased or decreased. The children's reading performance was registered by recording their voice with a microphone. Speech onset, the presented pseudoword, the presentation time of the pseudoword, and the voice of the subject were recorded by a computer. The experiment took no longer than 45 minutes.

#### 2.2.2 Statistics

The means of reading errors were compared by computing effect sizes (Hedges & Olkin, 1985; Borenstein et al., 2009).

Cohen $d=\frac{X_{1}-X_{2}}{\mathrm{Sw}}\mathrm{Sw}=\sqrt{\frac{(n_{1}-1)S_{1}^{2}+(n_{2}-1)S_{2}^{2}}{\left(n_{1}+n_{2}-2\right)}}$ and J = 1- $\frac{3}{4\;\mathrm{df}-1}$ is the Hedges correction factor for d; Hedges g = (d × J) where *df* are the degrees of freedom. *X*_1_ and *X*_2_ are the means and *S*_1_ and *S*_2_ are the standard deviations of the rate of reading mistakes. *n*_1_ and *n*_2_ are the number of values from which each mean value was calculated. In addition, means were compared using the Wilcoxon test.

Differences based on *p*-values were calculated using Fisher's exact test. For multiple comparisons, *p*-values were Bonferroni-Holm corrected. However, *p*-values were not interpreted within the framework of significance statistic. In accordance with the suggestions of Benjamin & Berger (2019), *p*-values between 0.05 and 0.005 were regarded as suggestive.

### 2.3 Results

The results of the pseudoword experiment are summarized in Table 1. The 7 children who were able to read only 3 letters simultaneously had a mean speech-onset latency of 1617.53 ms (SD = 449.20 ms). Four letters were recognized simultaneously by 17 children. The mean speech-onset latency was 1524.02 ms (SD = 558.51 ms). The ability to recognize 5 letters simultaneously was observed in 11 children. The mean speech-onset latency was 1635.96 ms (SD = 472.91 ms). Five children were able to recognize 6 letters simultaneously. Their speech-onset latency was 1579.45 ms (SD = 348.61). The number of letters a child could recognize simultaneously had no effect on the time the children needed from the beginning of the presentation of a pseudoword until the correct pronunciation (speech onset latencies). A comparison of the mean values of the speech onset latencies showed an effect size (Hedges’ g) between 0.13 (confidence interval: –0.107–0.366; confidence coefficient: 95%.) and 0.215 (confidence interval: 0.045–0.385). This implies that there was no notable difference between speech onset latencies.

**Table 1:** The number of letters (columns 2–5, from left to right), fixation times (first column on the left) and mean speech onset times (bottom row) at which 40 dyslexic children were able to read at least 95% of the pseudowords correctly

Fixation Time	Number of Letters Recognized			
	3 Letters	4 Letters	5 Letters	6 Letters
	Number of Subjects who Recognized >95% of the Pseudowords Correctly			
250 ms	3 (T:2; C:1)	5 (T:2; C: 3)	7 (T:3; C:4)	3 (T:2; C:1)
300 ms		2 (T:1; C:1)		
350 ms	1 (C)	2 (T:1; C:1)	3 (T:1; C:2)	
400 ms	1 (T)	3 (T:2; C1)	1 (T)	2 (C)
450 ms	1 (T)	5 (T:3; C:2)		
500 ms	1 (C)			
*Σ* subjects	7	17	11	5
Speech Onset Latency	X = 1617.53 ms	X = 1524.02 ms	X = 1635.96 ms	X = 1579.45 ms
	SD = 449.20 ms	SD = 548.51 ms	SD = 472.91 ms	SD = 348.61 ms

First column on the left: presentation times (i. e. fixation times) of the pseudowords; second column: number of subjects who were able to read 3-letter pseudowords within fixation times between 250 and 500 ms; third column: number of subjects who were able to read 4-letter pseudowords within fixation times between 250 and 500 ms; fourth column: number of subjects who were able to read 5-letter pseudowords within fixation times between 250 and 500 ms. Fifth column: number of subjects who were able to read 6-letter pseudowords within fixation times between 250 and 500 ms. Bottom row: means and standard deviations of speech onset latencies. Brackets indicate the number of children who belonged to the therapy group or control group: T: therapy group; C: control group.

Children who could only recognize 3 letters simultaneously (*n* = 7) read 140 pseudowords correctly if they were presented for a convenient fixation time. Further, out of 8 word lists consisting of 20, 3-letter words (160 words in total), only one word of each word list was read incorrectly. If 15 lists of 3-letter pseudowords (i.e. 300 pseudowords) were presented to children who could recognize 3 letters simultaneously for too short a fixation time, or if these children were offered 4-letter pseudowords, at least 2 pseudowords were read incorrectly in each list. A total of 136 (30.91%) 3- and 4-letter pseudowords were misread out of 440; they were presented for a fixation time between 250 ms and 500 ms. On average, 45.33% (SD = 18.48%) of the pseudowords in a list of 20, with a length of 3 letters, were read incorrectly. The mean fixation time was 325 ms (SD = 55.9 ms).

Seventeen children were able to recognize 4 letters simultaneously. They read 12 lists of 20 pseudowords each (i.e. 240 pseudowords) without errors. In another 8 lists of 20 pseudowords each, only one word was read incorrectly in each list. In total, out of 400 pseudowords 4 letters long and a fixation time suitable for the children, only 8 pseudowords were read incorrectly. In 34 lists consisting of 20 pseudowords each (i.e. 680 pseudowords), which had too short a fixation time, or were one letter longer than the number of letters the children could recognize simultaneously, at least 2 letters were misread in each of the 247 (36.32%) pseudowords. On average, 26.90% (SD = 15.43%) of the words in a list of 20 4-letter pseudowords were misread. There was a marked difference between the mean value of 3-letter pseudowords and the mean value of 4-letter pseudowords (Hedges' g = 1,134; Confidence Interval: 0.509–1,759; Confidence Coefficient: 95%). The mean fixation time was 328 ms (SD = 92.4 ms).

Eight lists of 20, 5-letter pseudowords each (i.e. 180 pseudowords) with a length of 5 letters were read without errors. In each of the 3 lists consisting of 20 pseudowords each, only one letter was read incorrectly. In each of the 3 lists consisting of 20 pseudowords each, only one word was read incorrectly if the pseudowords had a length of 5 letters. Thus, out of 140 pseudowords, 7 were read incorrectly. In 9 word lists consisting of a total of 180 pseudowords, 46 words (25.46%) were read incorrectly if these pseudowords had a length of 6 letters or were offered for a shorter fixation time than the required. On average, children (*n* = 11) who were able to simultaneously recognize 5 letters, misread a mean of 18.69% (SD = 13.21%) pseudowords in a list of 20 pseudowords. The difference between the mean rate of misread 4-letter pseudowords and the mean rate of misread 5-letter pseudowords was weak (Hedges’ g = 0.559; Confidence Interval: –0.042–1.076, Confidence Coefficient: 95%). The mean fixation time was 386.9 ms (SD = 114.4 ms).

**Fig.1:**
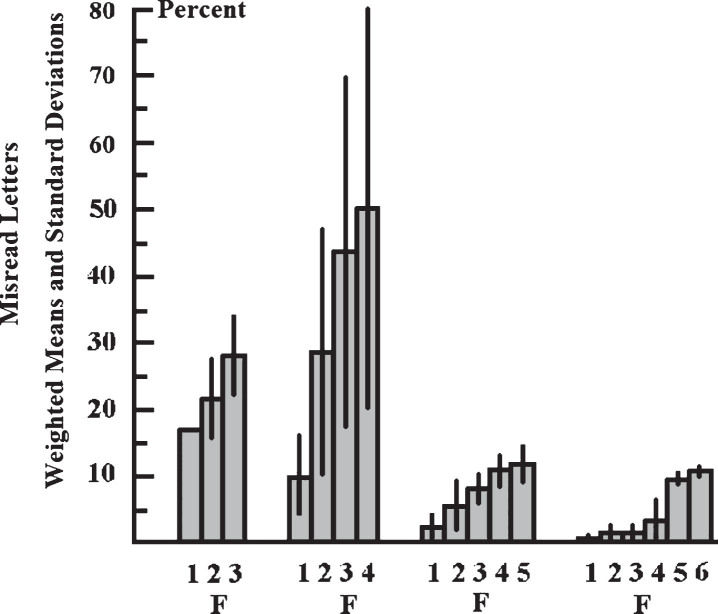
Shows the weighted mean values (columns) and the weighted standard deviations (bars) of misread letters at a certain position in pseudowords consisting of 3 to 6 letters. F: Letter that was at the fixation point. 1: first letter at the beginning of the word; 2: second letter from left; 3: third letter from left, etc. The letters were displayed between 250 and 500 ms. At different presentation times (e. g. 250 ms), a different number of pseudowords of a certain length were presented. However, the rate of misread letters was calculated over all different presentation times between 250 ms and 500 ms. Therefore, the rate (%) of the letters that were misread at a given presentation time was weighted with the sum of the letters that made up the misread pseudowords. The rate of misread letters increased from the first letter at the beginning of the word to the last letter at the end of the word regardless of the word length. A comparison of the weighted mean values with the Hedges'g effect size showed Hedges'g values between g = 0.107 and g = 0.15. This means that there was no effect of the position of letters in the pseudowords on the rate of misread letters.

Children (*n* = 5) who could recognize 6 letters simultaneously in 3 lists consisting of 20 pseudowords each, misread only one letter in each word list. In 4 lists consisting of 20 pseudowords each, which were presented with a shorter fixation time than the required, 14 pseudowords were read incorrectly. This implies that on average 12.14% (SD = 7.49%) of a list consisting of 20 pseudowords were read incorrectly. The examination of the influence of the length of pseudowords on the rate of misread letters, in a list of 20 pseudowords demonstrated a marked difference between the mean of misread 5-letter pseudowords and the mean of misread 6-letter pseudowords (Hedges’ g = 1.01; Confidence Interval: 0183–1.837, Confidence Coefficient: 95%). The mean fixation time was 458.3 ms (SD = 93.2 ms). Figure 1 shows the weighted mean frequencies and weighted standard deviations with which letters were read incorrectly at a certain position in 3- to 6-letter pseudowords. Exact values are listed in Table 2. The rate of misread letters increased from the first letter, at the beginning of the word, to the last letter, at the end of the word, regardless of the word's length. A comparison of the weighted mean values with the Hedges'g effect size showed Hedges'g values between g = 0.107 and g = 0.15. This means that there was no effect of the position of letters in the pseudowords on the rate of misread letters.

**Table 2:** First row: length of pseudowords. First column: positions of omitted letters in pseudowords

Position of Letter in the Word	3 Letter Words	4 Letter Words	5 Letter Words	6 Letter Words
First Letter	Xw = 16.67%	Xw = 10.03%	Xw = 3.48%	Xw = 0.51%
	SDw = 0%	SDw = 6.53%	SDw = 2.73%	SDw = 0.72%
Second Letter	Xw = 22.22	Xw = 26.99%	Xw = 6.52%	Xw = 1.01%
	SDw = 6,08%	SDw = 17.68%	SDw = 4.05%	SDw = 1.43%
Third Letter	Xw = 27.78%	Xw = 44.19%	Xw = 7.82%	Xw = 1.01%
	SDw = 6,08	SDw = 26.56%	SDw = 3.39%	SDw = 1%
Forth Letter		Xw = 49.82%	Xw = 11.09%	Xw = 4.04%
		SDw = 30.34%	SDw = 2.12%	SDw = 4.63%
Fifth Letter			Xw = 12.0%	Xw = 10.1%
			SDw = 1.67%	SDw = 0.35%
Sixth Letter				Xw = 11.6%
				SDw = 0.69%

Second to sixth columns: weighted means (Xw) and weighted standard deviations (SDw) of misread letters at a given position in pseudowords of a given length.

In addition, the percentages of misread letters at certain positions in the pseudowords were compared using Fisher's exact test. For 3-letter pseudowords, the difference between the frequency of misread letters at the 1st, 2nd or 3rd position within a pseudoword was *p* > 0.5 (Bonferroni-Holm corrected; Fisher's exact test). For 4-letter pseudowords, the difference between the frequency of reading mistakes at positions 1 and 2 within a pseudoword was *p* < 0.001 (Bonferroni-Holm corrected; Fisher's exact test). The difference between the frequency of reading mistakes at the 2nd and 3rd positions was *p* < 0.0008 (Bonferroni-Holm corrected; Fisher’s exact test). The frequency of reading mistakes at positions 1 and 3 within a pseudoword was *p* < 0.001 (Bonferroni-Holm corrected; Fisher's exact test). The difference between the frequency of reading mistakes at positions 3 and 4 within a pseudoword was *p* > 0.1 (Bonferroni-Holm corrected; Fisher's exact test). For 5-letter pseudowords the difference in the frequency of misread letters at the 1st and 2nd positions, the 2nd and 3rd positions, the 3rd and 4th positions, and the 4th and 5th positions was *p* > 0.1 (Bonferroni-Holm corrected; Fisher’s exact test). There were only a differences between the 1st and the 4th and the 1st and the 5th positions (Bonferroni-Holm corrected: *p* < 0.001; Fisher's exact test). For 6-letter pseudowords, there were only differences between the 1st and the 3rd (Bonferroni-Holm corrected: *p* < 0.031; Fisher's exact test) and the 2nd and the 6th positions (Bonferroni-Holm corrected: *p* = 0.0404; Fisher’s exact test).

The results show that the number of letters a pseudoword consists of and the time it takes to fixate it are crucial to whether or not a pseudoword is read correctly. Reading mistakes occur if readers try to recognize more letters simultaneously than the reader is able to recognize simultaneously and because the fixation time is too short. An extension of the fixation time and a reduction of the number of letters led to an improvement in pseudoword recognition. It can therefore be assumed that even when reading a text, an extension of the fixation time and a division of the text into segments containing only as many letters as a child can recognize simultaneously, leads to an improvement in the reading performance.

## 3 Experiment 2: Improvement of reading performance by a compensatory reading therapy

This therapy experiment investigated if a child’s ability to read a normal text improves when:

(1) the child only attempts to simultaneously recognize words or word segments consisting of no more letters than s/he is able to recognize simultaneously,
(2) the amplitudes of the reading saccades do not exceed the number of letters the child is able to recognize simultaneously,
(3) the child fixates the word segments for the time interval needed (adequate fixation intervals), and
(4) the time interval between the onset of the presentation of the word and the onset of the pronunciation of the word is sufficiently long (adequate speech onset latency).

How many letters the child is able to recognize simultaneously, how big the eye movement amplitudes should be, how long the child has to fixate a word to recognize a given number of letters, and how long the time interval between the onset of the word's presentation and the onset of its pronunciation (speech onset latency) should be, is adjusted according to the results of the pseudoword experiment. Thus, the reading strategy which the subjects learn in the therapy experiment presupposes the result of the compensatory pseudoword experiment. To adopt an adequate reading strategy in the therapy experiment, children learned to:

(1) divide the text into word segments that are not larger than the number of letters the child can recognize simultaneously,
(2) fixate these words or word segments for the appropriate time interval,
(3) start to pronounce the words or word segments only after an appropriate time interval, and
(4) execute eye movements of an amplitude matching the length of the words or word segments whose letters could be recognized simultaneously (adequate reading saccades).

### 3.1 Patients

All patients who had participated in Experiment 1 participated in Experiment 2.

### 3.2 Methods

#### 3.2.1 Procedure

The children were assigned to the therapy group (20 children) or to the control group (20 children) according to their ability to read letters simultaneously. After each pseudoword experiment the amount of letters a child could recognize simultaneously was known. Children who could recognize the same number of letters simultaneously were assigned to the therapy group or the control group in such a way that there was approximately the same number of children in each group. If several children had the same ability to recognize a certain number of letters simultaneously, the children were assigned to the therapy group and control group in such a way that there were approximately the same number of children in each group who needed the same fixation time. Children who had almost the same ability to recognize a certain number of letters simultaneously and needed the same fixation time were assigned to the therapy group or the control group in such a way that in both groups there were approximately the same number of children who had almost the same age. Thus, the therapy group and the control group were similar in the ability to read letters simultaneously, and in the fixation time they needed to read a given number of letters simultaneously. Table 1 shows the distribution of the children in the therapy group and the control group (mean age in the therapy group: 121.8 months; SD 13.77 months; mean age in the control group: 124.05 months SD: 17.96 months).

The children were sitting in front of a monitor. The distance between the eyes and the monitor was 40 cm. The words were black (luminance of 4 cd/m²; altitude 14 mm; space between types: 4 mm) on a background of 68 cd/m². Fixation of the word segments and saccadic eye movements were recorded using an infrared eye-tracking system (IRIS eye tracker; sampling rate: 500 Hz). Eye movements were monitored online, stored, and analyzed online and offline. Heads were stabilized with forehead rest and side head restraints to minimize movements while reading.

Only the therapy group participated in the compensating reading therapy in which they learned a new reading strategy using the Celeco Software-Package for the Diagnosis and Therapy of Dyslexia (Werth et al., 2006, 2019). In this reading therapy, a yellow fixation mark indicated the point within each word or word segment to which the gaze was to be directed. A green cursor (segment cursor) to the left and/or right of the yellow fixation mark indicated which letters in the word segment were to be read simultaneously together with the letter at the location of the yellow fixation mark. The yellow and green marks indicated which adjacent letters in a word or word segment should be read while the eyes fixated the yellow fixation mark. The subjects were to read the text aloud such that reading errors could be recognized immediately by the therapist. Whenever a word segment was recognized, the next word segment was shown. Then the yellow fixation mark was moved to the middle letter of the next word or word segment, indicating the goal of the saccade, (i.e. the location where the gaze should be directed when the next word segment is read). A green cursor (segment cursor) to the left and/or right of the yellow fixation mark again showed which letters of the newly shown word or word segment were to be read while the eyes were directed to the shifted yellow fixation mark. The fixation mark and the segment cursor moved from one word segment to another as they were to be read in succession. The text to the right of a word or word segment to be read was not shown on the monitor to prevent the child from terminating fixation of a word or word segment by exerting a premature saccade before the necessary fixation time had elapsed. The next word segment to be read was shown only after the previous word segment had been recognized. An acoustic signal was presented 2 sec. after the yellow and the green cursors had been moved to the new segment to be read. The acoustic signal indicated when the subject was allowed to pronounce the word segment. If this did not improve reading, the sound signal was given one second after the appearance of the word segment.

Half of the children in the therapy group read the first part of cards 3, 4 and 5 of the ZLT before the therapy session and the second part after the therapy session. The other half of the children in the therapy group read the second part of the cards 3, 4 and 5 before the therapy and the first half after the therapy. Half of the children in the control group read the first part of cards 3, 4 and 5 of the ZLT first and the second part later. The other half of the controls read the second part of the cards 3, 4 and 5 first and the first part later. The controls read the same text for the same amount of time as the children in the therapy group during the therapy session after having read one half of the ZLT and before reading the other half of the ZLT. The controls received no therapy while reading the same text. Card 3 consisted of 89 words (405 letters), card 4 consisted of 92 words (474 letters), and card 5 consisted of 80 words (452 letters). The first part of cards 3, 4 and 5 consisted of 129 words and 665 letters, and the second part consisted of 132 words and 666 letters. The experiment took no longer than 45 minutes.

#### 3.2.2 Statistics

The effect of the therapy compared to controls without therapy was calculated by computing effect sizes (Hedges & Olkin, 1985; Borenstein et al., 2009). Hedges′g = (d × J) $d=\frac{X_{r1}-X_{r2}}{\mathrm{Sw}}$ $\mathrm{Sw}=\sqrt{\frac{(n_{r1}-1)S_{r1}^{2}+(n_{r2}-1)S_{r2}^{2}}{\left(n_{r1}+n_{r2}-2\right)}}$ and J = 1- $\frac{3}{4\;\mathrm{df}-1}$ where *df* are the degrees of freedom and J is a correction factor for d. *X*_*r*1_ is the mean and *S*_*r*1_ is the standard deviation computed from the result of the reading test before therapy. *X*_*r*2_ is the mean and *S*_*r*2_ the standard deviation computed from the result of the reading test after therapy. *n*_*r*1_ is the number of subjects participating in the reading test before therapy and *n*_*r*2_ is the number of subjects participating in the reading test after therapy. The formula above also applies to the control experiment. Then, the number of subjects *n*_*r*1_ is replaced by the number of subjects *n*_*u*1_ in the first reading test of the control experiment and the number of subjects *n*_*r*2_ is replaced by the number of subjects *n*_*u*2_ in the second reading test of the control experiment. The mean of reading errors *X*_*r*1_ is replaced by the mean of reading errors *X*_*u*1_ in the first reading test of the control experiment. The mean of reading errors *X*_*r*2_ is replaced by the mean of reading errors *X*_*u*2_ in the second reading test of the control experiment. The standard deviation *S*_*r*1_ is replaced by the standard deviation *S*_*u*1_ of the first reading test of the control experiment. The standard deviation *S*_*r*2_ after the reading therapy in the therapy group is replaced by the standard deviation *S*_*u*2_ of the second reading test in the control experiment.

*P*-values were also calculated by the Wilcoxon-test for the comparison between the mean reading errors before and after the therapy and for the comparison of the mean reading errors between the first and second reading test without therapy. However, these *p*-values should not be interpreted as significant or insignificant and they should not be used to accept or reject a hypothesis.

### 3.3 Results

In the therapy experiment, the children misread a mean of X = 11.95 (SD = 6.12) words on the first part of cards 3, 4, and 5 of the ZLT before, and a mean of X = 5.1 (SD = 2.83) words on the second part of these cards after the reading therapy. The therapy effect on reading performance was very high (Hedges’g = 1.4; confidence interval: 0.742–2.132; confidence coefficient: 95%; Wilcoxon-test: *p* < 0,00001), whereas there was no effect between the rate of reading mistakes in the first and the second reading test in the control group. The controls misread a mean of X = 12.1 (SD = 8.19) words when reading the first part of cards 3, 4, and 5 of the ZLT. They misread a mean of X = 13.9 (SD = 8.04) words when reading the second part of the ZLT (Hedges’g = 0.222; confidence interval: –0.4–0.844; confidence coefficient: 95%; Wilcoxon-test: *p* > 0.1). The rate of reading mistakes increased somewhat in the controls when reading the second part of the ZLT.

## 4 Experiment 3: The investigation of eye movements before and after compensatory reading therapy

### 4.1 Patients

Eye movements were recorded in all 20 children in the therapy group before and after the compensatory reading therapy and in all 20 children in the control group when reading card 4 of the ZLT.

### 4.2 Methods

#### 4.2.1 Procedure

Eye movements were recorded while the children were reading card 4 of the ZLT using an infrared-light-reflecting, eye-tracking system (IRIS eye tracker; Bablok-b-scope, sampling rate: 500 Hz, resolution: 2 min arc). Heads were stabilized with a forehead rest and side head restraints to minimize movements while reading. Since this was not very comfortable, the eye movements were recorded only while reading card 4 of the ZLT. The first part of card 4 consisted of 46 words (231 letters), the second part of card 4 also consisted of 46 words (243 letters). Half of the children in the therapy group read the first part of card 4 of the ZLT before and the second part of card 4 after the therapy session. The other half of the children in the therapy group read the second part of the card 4 before and the first half after therapy. Half of the children in the control group read the first part of card 4 first and the second part later. The opposite occurred for the other half of the controls. They read the second part of the card 4 first and the first part later. There was no visual or acoustic signal that could support the reading performance during eye movement recording.

#### 4.2.2 Statistics

The effect of the therapy on eye movements was computed using Hedges' g effect size as described in section 3.2.2. In addition, *p*-values were computed using the Wilcoxon test.

### 4.3 Results

**Fig.2:**
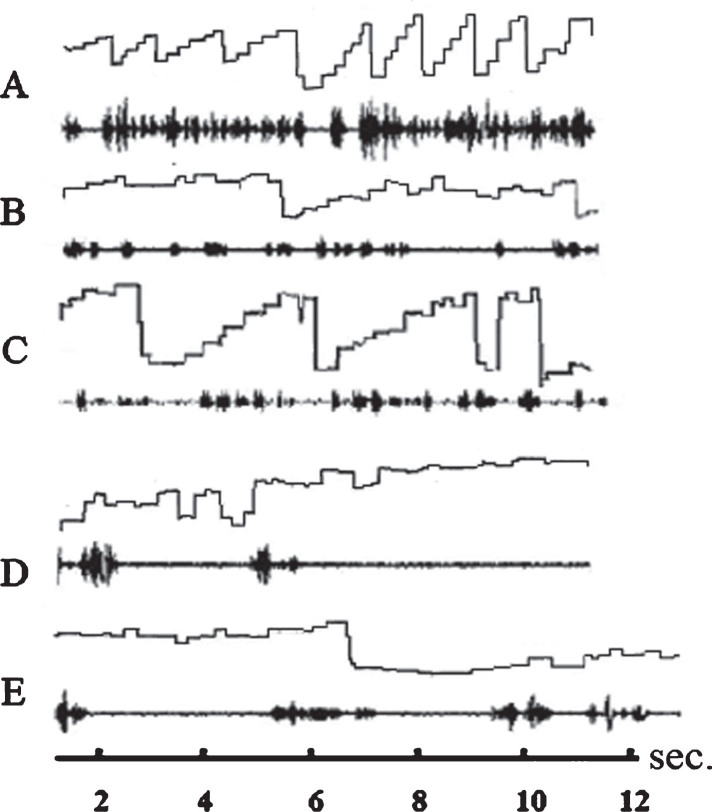
Eye movements and speech recording of three subjects while reading a text from the ZLT. Abscissa: time axis; ordinate: amplitude of eye movements. Ascending line: eye movement to the right; descending line: eye movement to the left. The spectrogram of the reader’s language is displayed below each graphic representation of eye movements. A: ideal staircase eye movements of a good reader (not included in the study). B: eye movements and speech spectrogram of a dyslexic reader before therapy. The subject is reading slowly with only few staircase-like eye movements and many eye movements against the reading direction (reversions). C: eye movements and speech spectrogram of the same subject after therapy. Sequences during which the subject performs staircase-like eye movements are more frequent than before therapy but there are still many reversions. D: eye movements and speech spectrogram of a dyslexic subject who is reading very slowly. The subject speaks only after having performed a series of searching eye movements. E: The subject performs more staircase-like eye movements but executes still many reversions. The speech is much more fluent. All subjects reduce reading mistakes after therapy by almost two thirds.

When the children in the therapy group and in the control group read the ZLT text, none of the dyslexic readers executed only staircase-like eye movements in the reading direction like good readers do (Fig. 2A). All readers performed numerous single regressive saccades or a series of step-like regressions against the reading direction (Fig. 2B-E). The rate of saccades to the right and left increased after therapy. On average, children executed X = 63.35 (SD = 18.97) saccades to the right, and X = 26.7 (SD = 12.50) saccades to the left before therapy. After therapy, the children executed X = 102 (SD = 28.13); saccades to the right and X = 48.6 (SD = 20.44) to the left. If one compares the mean rate of eye movements to the right before and after therapy, Hedges'g effect size shows that therapy had a very strong effect on eye movements to the right (Hedges'g = 1.785; confidence interval: 1.052–2.518; confidence coefficient: 95%; Wilcoxon-test: *p* < 0.0001) and a strong effect on the rate of saccades to the left (Hedges'g = 1.293; confidence interval: 0.611–1.974; confidence coefficient: 95%; Wilcoxon-test: *p* < 0.001). There was no difference beween the rate of eye movements to the right (Hedges'g = 0.066; confidence interval: –0.0686–0.554; confidence coefficient: 95%; Wilcoxon-test: *p* > 0.1) and those to the left (Hedges'g = 0.198; confidence interval: –0.819–0.423; confidence coefficient: 95%; Wicoxon-test: *p* > 0.1) in the control group. Controls executed a mean of X = 88.25 (SD = 36.88) saccades to the right and a mean of X = 32.9 (SD = 18.12) to the left when reading one half of card 4 first. They executed a mean of X = 85.5 (SD = 45.50) saccades to the right and a mean of X = 29.5 (SD = 16.18) saccades to the left when reading the other half of card 4 later.

## 5 Meta-analysis and causes of dyslexia

### 5.1 Patients

A total of 256 children aged between 7 and 16 years who participated in the earlier studies (Werth, 2006, 2018; Klische, 2007) were included in the meta-analysis. In addition, 40 children (23 boys and 17 girls) aged between 8 and 15 years who participated in the present study on the effect of the compensatory reading therapy on reading performance were also included in the meta-analysis. All children who participated in the earlier studies (Werth, 2006, 2018; Klische, 2007) and those who participated in the present one were diagnosed as dyslexic according to the Zuerich Reading Test (Linder & Grissemann, 2000). None of the children were above the 15 percentile in the ZLT reading test. The children were native German speakers and right-handed. They had no neurological, psychiatric, visual, or auditory deficits, and no speech disorders. The children's IQs were within the normal range. They were second-to-sixth graders who knew all individual letters, had approximately the same lessons in reading, and were expected to read fluently, but were far behind the required reading ability.

### 5.2 Methods

#### 5.2.1 Procedure

In all studies included in the meta-analysis, the pseudoword experiment and the compensatory reading therapy were performed in the same way as in the experiments 1 and 2 described previously using the Celeco Software Package for the Diagnosis and Therapy of Dyslexia (Werth et al., 2006, 2019).

#### 5.2.2 Statistics

The Hedges’g effect size was calculated as previously described in paragraph 3.2.2.

The summary effect was calculated within the framework of a fixed-effect meta-analysis. The result of each experiment is weighted with a factor W_i_. W_1_ is the weight that is assigned to the result of the first experiment, and W_i_ is the weight that is assigned to the result of the ith experiment.

$W_{i}=\frac{1}{\mathrm{Vgi}}$ whereby V_gi_ = J^2^ × V_di_ and $V_{\mathrm{di}}=\frac{n_{r1}+n_{r2}}{n_{r1}+n_{r2}}+\frac{d_{i}^{2}}{2(n_{r1}^{2}+n_{r2}^{2})}$

*d*_*i*_ is the Cohen d value computed from the result of the ith experiment.

$$d\;=\;\frac{X_{r1}-X_{r2}}{\mathrm{Sw}}\;\mathrm{Sw}\;=\;\sqrt{\frac{(n_{r1}-1)S_{r1}^{2}+(n_{r2}-1)S_{r2}^{2}}{\left(n_{r1}+n_{r2}-2\right)}}$$

The weighted mean Hedges summary effect computed from i experiments is $G=\frac{\sum_{i=1}^{3}\mathrm{Wiyi}}{\sum_{i=1}^{3}\mathrm{Wi}}$ whereby W_i_Y_i_ = W_i_ × g_i_. i = experiment 1, ...  , experiment q; n_r1_= number of experimental subjects in the reading test before therapy, n_r2_= number of subjects in the reading test after therapy. The same applies to the number of subjects n_u1_ in the first reading test of the control experiments and the number of subjects n_u2_ in the second reading test in the controlexperiments.

In addition *p*-values were calculated using the Wilcoxon-test for the comparison between the mean reading errors before and after the therapy, and for the comparison of the means of reading errors between the first and the second reading test without therapy. However, these *p*-values are not interpreted as significant or insignificant, and they are not used to accept or reject a hypothesis.

### 5.3 Results

The results of the meta-analysis of the three previous studies (Werth, 2006, 2018; Klische, 2007) and the present therapy study are shown in Table 3. In each of the four studies, a compensating reading therapy was performed, in which the words to be read were fixated at the correct location, the number of letters to be recognized simultaneously was reduced, the fixation time and speech onset latency was extended and the saccade amplitudes were adapted to the number of letters that could be read simultaneously. These measures led to a reduction in reading errors by almost two thirds in all studies. The therapy showed a very high summary effect of Hedges’ G = 1.72 (confidence interval: 0.006–2.97; confidence coefficient: 95%) in all four studies.

**Table 3:** First column on the left: studies included in the meta-analysis and the number of subjects tested

Study		Mistakes	Mistakes	Cohen d	Hedges g	Conf. Int.	*p*-value
		Mean%	SD%			Con Coeff = 95%	Tests
Werth 2006	Before Therapy	16.05	6.05	2.044	2.021	1.561–2.528	Wilcoxon
	After Therapy	6.13	3.24				*P*≤0.0001
*n* = 68 Children	Controls 1	11.07	5.40	0.314	0.310	–0.138–0.82	Wilcoxon
	Controls 2	13.24	7.19				*P*≥= 0.2
Klische 2007	Before Therapy	7.68	8.41	1.714	1.699	1.226–2.203	*t*-Test
	After Therapy	3.15	5.0				*P*≤0.0001
*n* = 88 Children	Controls 1	6.71	7.77	0.19	0.188	0.286–0.667	*t*-Test
	Controls 2	7.39	10.80				*P*≥0.119
Werth 2018	Before Therapy	14.8	6.14	1.734	1.721	1.275–2.194	Wilcoxon
	After Therapy	6.12	3.52				*P*≤0.00001
*n* = 100 Children	Controls 1	12.47	6.39	0.109	0.108	–0.284–0.501	Wilcoxon
	Controls 2	13.20	7.02				*P*≥0.1
Werth Present Study	Before Therapy	11.95	6.12	1.437	1.4	0.742–2.132	Wilcoxon
	After Therapy	5.1	2.83				*P*≤0.00001
*n* = 40 Children	Controls 1	12.1	8.19	0.109	0.108	–0.4–0.8	Wilcoxon
	Controls 2	13.9	8.04				*P*≥0.1

Third column: mean reading errors (percent) before and after therapy and mean of the first (controls 1) and second (controls 2) examination of the control group in a given study. Fourth column: standard deviation of reading errors (percent) before and after therapy and standard deviation of the first (controls 1) and second (controls 2) examination of the control group in a given study. Fifth and sixth columns: Cohen d-values and Hedges’g-values of the therapy- and the control studies. Seventh column: confidence intervals. In addition, *p*-values of differences between reading mistakes before and after therapy and between the first and the second reading test in the control-studies are reported in the eighths column. The *p*-values are only added for the sake of completeness as they were calculated in earlier studies within the framework of a *p*-value-based significance statistic. However, the *p*-values are not interpreted as criteria for significant differences according to the statistical specifications of the American Statistical Association.

All four studies display a causal relationship between (a) the presence of given impairments and a deterioration of reading performance, and (b) between the compensation of these impairments and an improvement of reading performance (for a scientific definition of the concept of cause see: Mackie 1965; Spohn 1980, 2006; Spirtes et al. 1992; Lewis 2000; Pearl 2003; 2000–2018; Werth 2010; Pearl et al. 2016; Werth 2019). If the impairments were compensated, then reading performance improved. The meta-analysis revealed the following causes for a reading deficiency:

1) An impaired ability to simultaneously recognize a sequence of letters within a word along with the attempt to recognize more letters simultaneously than the reader was able to,
2) a prolonged fixation time required to recognize a sequence of letters within a word, along with a premature new saccade to the next word or word segment,
3) saccade amplitudes that exceed the number of letters that the reader is able to recognize simultaneously, and
4) an extended time needed to retrieve the phonemes corresponding to the graphemes from memory along with a premature onset of the pronunciation of the word, and a premature onset of a saccade to the next word or word segment.

## 6 Discussion

### 6.1 The role of simultaneous recognition, the field of attention, and the crowding effect

The paper aims to show that reading mistakes occur: if a reader tries to recognize more letters simultaneously than s/he is able to, if amplitudes of saccades are not determined by the reader's ability to simultaneously recognize a sequence of letters, if the reader does not adhere to the fixation interval that s/he needs to recognize a sequence of letters, and if a reader pronounces a word before the phonemes have been retrieved correctly from memory. This is examined in the pseudoword experiment (experiment 1). If pseudowords that are made up of only so many letters as the subjects are able to recognize simultaneously, if the subjects fixate the pseudowords for a sufficiently long time, and if the subjects pronounce the words only after the phonemes have been retrieved correctly from memory, even dyslexics can read at least 95% of the pseudowords correctly. The aim of the pseudowort experiment was to find the right number of letters the words should have, to find the adequate length of the fixation times, and the adequate length of the speach onset times, such that the subjects can recognize at least 95% of the pseudowords correctly. In the pseudoword experiment the computer establishes the appropriate conditions for the subjects to correctly recognize 95% of the pseudowords by presenting pseudowords of an appropriate length for an appropriate fixation time, and by indicating when the subject is allowed to pronounce the pseudoword. However, the subjects do not yet learn a reading strategy that allows them to establish these conditions themselves. The subjects only learn such a reading strategy in the compensatory reading therapy (experiment 2). This reading therapy incorporates the results of the pseudoword experiment. In this reading therapy the subjects learn how to split the text into segments containing no more letters than they can recognize simultaneously, which saccadic amplitudes they should perform, how long they should extend the fixation and the speech onset times, according to the results of the pseudoword experiment. Thus, the pseudoword experiment is used for diagnostics and the compensatory reading therapy is used for treatment. As both the pseudoword experiment and the therapy, each constitute an intervention, the present approach can be regarded as a “dual intervention approach”. After computer based training of less than 30 minutes the rate of reading mistakes dropped in all studies by almost 60%.

The ability to recognize multiple letters simultaneously was investigated when reading pseudowords, as words that occur in normal language are not suitable for this purpose. Words in the normal language can be assumed even if only a few letters of the word have been recognized. A reduced ability to recognize a succession of letters simultaneously, which is a cause of dyslexia, can also not be regarded as the consequence of a lack of knowledge of the grapheme-phoneme correspondence. A reduced ability to recognize a succession of letters simultaneously exists even if the subjects have learned the grapheme – phoneme correspondence and if the memory of the grapheme-phoneme correspondence is unimpaired. Furthermore, a reduced ability to recognize a succession of letters simultaneously cannot be only attributed to a narrowing of the field of attention (Facoetti et al., 2000; Carrasco et al., 2002; Godijn & Theeuwes, 2003; Gersch et al., 2004; Valdois et al., 2004; Bosse et al., 2007; Baldauf & Deubel, 2008; Franceschini et al., 2012), or to an enhanced crowding effect (Spinelli et al., 2002; Martelli et al., 2009; Whitney & Levi, 2011; Gori & Facoetti, 2015) or to unusual foveal or parafoveal processing (Geiger & Lettvin, 1987; Rayner et al., 1989; Atkinson, 1997; Lorusso et al., 2004; Gori & Facoetti, 2015). If a narrowing of the field of attention was the cause of reading mistakes, most errors would be expected to occur at the beginning and the end of the pseudowords. However, this is contradicted by the results of the present study, which shows that the rate of misread letters is greater in the middle than at the beginning of the pseudowords. This result of the pseudoword experiment also shows that reading errors cannot be only attributed to an increased lateral masking effect. Letters at the right end of the pseudowords are not masked by other letters on both sides, whereas letters in the middle of pseudowords are masked by other letters to the left and to the right (crowding effect). Nevertheless, the subjects misread more letters on the right end of the pseudowords than in the middle irrespective of the length. This supports the assumption that poor reading performance is not exclusively due to a stronger crowding effect in dyslexic readers (Martelli et al., 2009; Spinelli et al., 2002; Whitney & Levi, 2011; Gori & Facoetti, 2015).

Compensatory reading training cannot be regarded as spatial-attention training. In the compensatory reading therapy, the expansion of the field of attention was not trained, and the training was not limited to the location of the focus of attention. It is crucial for the training that the amplitudes of the saccades that a reader has to perform are determined by the number of letters that a reader can recognize simultaneously within a fixation phase. For example, if a reader can only recognize 4 letters simultaneously, but executes a saccade over 6 letters, s/he will necessarily overlook 2 letters. In this case s/he overlooks 2 letters even if s/he can extend the field of attention over 6 letters. The area that comprises the number of letters a reader can recognize simultaneously may be smaller than the field of attention. Therefore, a reader may recognize a smaller number of letters than the field of attention comprises. Poppelreuter (1917) was the first to describe the ability of simultaneous recognition and its disorders in brain-damaged patients, and recognized this ability as a brain capacity of its own. Werth (2001) described the role of a diminished ability to simultaneously recognize several letters in a word and its connection with the saccade amplitudes, necessary for reading. The role of limited simultaneous recognition in the development of reading disorders has been experimentally demonstrated in several studies (Werth, 2006; Klische, 2007; Werth, 2018).

### 6.2 Do eye movements cause dyslexia?

The question of whether or not eye movements that deviate from the norm during reading can cause a reading disorder remains controversial (Pavlidis, 1981, 1985; Rayner, 1985; Hyönä & Olson, 1995; Biscaldi et al., 2000; Fischer & Hartnegg, 2000; Hutzler et al., 2006; Stein, 2018a, b; Blythe et al., 2018). It has been argued that the irregular eye movements frequently found in subjects with a reading disorder can also occur in good readers and that poor readers can also show normal eye movements.

Good readers perform a series of rapid eye movements (saccades) in the reading direction - in most languages from left to right (Fig. 2A). Dyslexic readers typically perform not only eye movements in the reading direction but also numerous eye movements against the reading direction (reversions) (Fig. 2B-E). However, some dyslexic readers also perform occasionally staircase eye movements in the reading direction, which dominate among good readers (Fig. 2C). Nevertheless, numerous reading errors occur in phases in which dyslexic readers perform staircase eye movements. Despite staircase eye movements the saccades are often greater than the number of simultaneously recognized letters, and the fixation times and/or the speech onset times are too short which leads to reading errors.

The text cannot be recognized during a saccade, but only when the eyes are at rest. Therefore, each saccade is followed by a fixation phase during which the eyes are focused on a word or word segment. During the fixation phase, several letters are read almost simultaneously. The ability for simultaneous recognition requires a brain capacity of its own, which can be reduced independently of other brain capacities. Depending on the reading speed, a saccade to the next word or word segment occurs after approximately 250 ms. When reading accurately, whereby all letters are recognized, a recognized word segment connects immediately to the next word segment, without a gap of unrecognized letters between recognized word segments. During fast, inaccurate reading, the amplitude of saccades may exceed the number of letters that can be recognized simultaneously. Then gaps of unrecognized letters occur between successive word segments or successive words. These gaps must be filled by assuming from the text context, as to which letters were overlooked.

Even readers with normal reading capabilities may exert eye movements against the reading direction. This is done, for example, to check whether a word segment that has already been read has been read correctly. Differences between poor and normal readers’ eye movement patterns were found in reading and non-reading tasks (Pavlidis, 1981; Biscaldi et al., 1994; Eden et al., 1994; Fischer et al., 1993; De Luca et al., 1999) whereas, no such differences were found in other eye movement studies (Adler-Grinberg & Stark, 1978; Olson et al., 1983; Stanley, 1983). While some researchers assume that irregular eye movements are a cause for reading deficiencies (Stein, 2018a, b; Stein et al., 1987), others assume that they are a consequence of other disorders that impair the reading process (Rayner, 1985; Hutzler et al., 2006; De Luca et al., 1999; Blythe et al., 2018). However, comparison between eye movements of poor readers and those of normal readers does not allow us to conclude whether abnormal eye movements are the cause or the consequence of reading problems.

Already the distribution of visual acuity in the visual field shows that eye movements resulting in inappropriate fixation lead to reading errors. Visual acuity is highest in the fovea and drops dramatically towards the periphery. Therefore, the most advantageous fixation strategy for reading is by fixating in the middle of the word (O’Regen, 1981; O’Regan & Lévi-Schoen, 1997). Then, as many letters as possible are displayed in the region with the highest visual acuity to both sides of the center of the fovea. If the gaze is directed to the beginning of the word, the word extends further into the right visual half field. Then, letters to the right end of the word fall into an area in which the visual acuity is no longer sufficient to recognize those letters. As a result, letters on the right end of the word cannot be recognized. The same occurs when the eye is directed to the right end of a word. Then, letters at the beginning of the word extend into an area that has too little visual acuity to recognize them, with the consequence that they are not accurately identified.

The amount of time that the gaze is directed toward a word and the time at which a saccade is initiated to the next word or word segment, is also decisive for recognizing the letters in the word or word segment. Earlier studies have already shown that dyslexics have longer fixation times than normal readers (Hari et al., 1999; De Luca et al., 2002; Kim et al., 2014; Al Dahhan et al., 2016). For a word to be read correctly, it is necessary for the word to be fixated for a sufficiently long time before executing a saccade to the next word (Werth, 2018). The fixation times needed to recognize a given number of letters simultaneously, differs among readers (Werth, 2016, 2018; Klische, 2007). If readers stop fixating before the end of the required fixation time when reading a word and execute a premature saccade to the next word, not all letters in the word that has been read can be recognized reliably.

A prerequisite for error-free reading is that the saccade amplitude matches the number of letters that a reader can recognize simultaneously. If a reader fixates words or word segments correctly such that they follow one another without a gap between them, it does not matter whether a reader executes eye movements to the left or to the right before s/he fixates on the next word or word segment to be read. If irregular saccades directed in any direction occur before or after fixating on the correct locations, and before or after fixating for sufficiently long intervals, the reading may slow down because time is wasted until the next word segment is fixated. Saccades directed to the left may make it more difficult to find the target for the subsequent fixation phase on the word to be read next. However, such irregular eye movements do not necessarily lead to reading mistakes.

However, reading errors occur if a sequence of casual searching eye movements across multiple words is executed and successive words or word segments are not fixated at the correct position, if word segments that contain a given number of letters that can be recognized simultaneously do not follow each other without gaps, and if the fixation times and the times required to retrieve the phonemes are not adhered to.

### 6.3 Eye movements that are the consequence of reading impairments

Readers who try to recognize more letters simultaneously than they can, inevitably make reading mistakes. The same applies if a word or word segment has been fixated for too short a time to be reliably recognized. If readers are uncertain whether they have read words or word segments correctly, they often execute eye movements to the left to re-fixate a word that had already been read. These reversions are the consequence of an insufficient recognition of words or word segments.

The same applies if words or word segments are pronounced before the phonemes corresponding to the sequence of letters recognized have been completely retrieved from memory. If the retrieval is not yet complete after the eyes have already moved to the following words or word segments, and a reading mistake occurs, readers often notice this and correct themselves. This often results in regressions to the words or word segments that have just been read. Uncertainty as to whether or not the text has been read correctly can, depending on the degree of difficulty of the text, occur in good readers as well as in dyslexic readers. However, dyslexic readers who make many reading mistakes because they start to pronounce the word before the phonemes have been retrieved correctly from memory will often re-fixate the already read words. Therefore, reversions may also occur due to premature pronunciation.

The result of the present study shows that after therapy, the rate of eye movements to the right increased. The increased rate of saccades to the right may be due to smaller eye movements as the readers divided the text into smaller segments. It can be assumed that this contributed to the improvement of reading performance. The rate of saccades to the left also increased, although the rate of reading errors decreased considerably. While some readers performed a series of staircase-like eye movements to the right without reversions after therapy other readers still performed numerous eye movements to the left afterwards. It can be assumed that after 20 minutes of therapy the eye movement pattern did not change fundamentally in many readers. However, even in readers who performed numerous reversions after therapy, these reversions did not prevent the improvement of reading performance if the word segments were fixated for a sufficiently long time and recognized word segments followed one another without a gap between them. Therefore, an increase of reversions does not necessarily prevent the improvement of reading performance.

### 6.4 P-values, reproducibility, and meta-analysis

The meta-analysis of the four studies (Table 3) shows that the compensatory reading therapy had a reproducible effect with extraordinarily high effect sizes after a single therapy session and that the results are reproducible. Since all four studies are different samples from all children with dyslexia, the result of each sample may be due to a sampling error that does not accurately reflect the effect of a treatment in all children. Therefore, the corrected summary effect size of Hedges' G was computed from all four studies to better estimate the true effect size. The extraordinarily high summary effect size of Hedges G = 1.72 substantiated the strong therapy effect. No other reading therapies showed a similar effect size despite many months of therapy (Galuschka et al., 2014).

The statistics for meta-analysis is based on effect sizes instead of *p*-values and significance criteria. For many decades, the decision on whether or not to accept or reject an experimental result was based on a *p*-value smaller than a significance limit (usually *p* < 0.05). Such a significance criterion has been criticized by statisticians for many years (Gigerenzer, 2004; Joannidis, 2005; Wasserstein, 2016; Wasserstein & Lazar, 2016; Benjamin et al., 2018; Billheimer, 2019; Wasserstein et al., 2019), without being accepted in medicine or psychology. The American Statistical Association came to the conclusion that, “Smaller *p*-values do not necessarily imply the presence of larger or more important effects, and larger *p*-values do not imply a lack of importance or even lack of effect  ...  ”, that “ ...  the widespread use of “statistical significance” (generally interpreted as “*p*≤0.05”) as a license for making a claim of a scientific finding (or implied truth) leads to considerable distortion of the scientific process ...  ” and that “ ...   a *p*-value, or statistical significance, does not measure the size of an effect or the importance of a result.” (Wasserstein, 2016). Therefore, *p*-values should be replaced by effect sizes and confidence intervals (Thompson, 1993; Lambdin, 2012).

Objections also come from a lack of reproducibility of scientific results (Gigerenzer, 2004; Joannidis, 2005; Wasserstein, 2016; Wasserstein & Lazar, 2016; Benjamin et al., 2018; Billheimer, 2019). Wasserstein (2016) states that “ ...  the statistical community has been deeply concerned about issues of reproducibility and replicability of scientific conclusions.”

The lack of reproducibility of psychological research results has also been confirmed experimentally (Joannidis, 2005; Klein, 2018). It has been shown that out of 28 peer-reviewed and accepted psychological studies, 16 were not reproducible at all and 5 showed a clearly weaker result (Klein et al., 2018). A lack of reproducibility in the treatment of reading disorders may be attributed to an inappropriate statistical analysis used to show that a result is significant even though there is no therapeutic effect. Another source of irreproducibility exists if subjects have not been diagnosed with sufficient accuracy and therefore have different types of reading disorders. As demonstrated by the pseudoword experiment and the compensatory therapy experiment, the ability to recognize several letters simultaneously may be very different among readers. While some readers can only recognize 3-letter words simultaneously, this ability is undisturbed in others (Table 1). Some readers have reading disorders because they try to recognize more letters simultaneously than they can. Others don’t try to recognize more letters simultaneously than they can, but execute saccades that are greater than the number of simultaneously recognized letters. Other readers do not adhere to the required fixation times and initiate saccades too early. Many readers already start pronouncing before the sequence of sounds has been correctly retrieved from memory.

The usual investigation of a therapy effect is to compare a group of dyslexics before and after therapy with a control group which received no therapy. If the subjects in the therapy group and the ones in the control group have completely different types of reading disorders, the two groups are not comparable. Before the patients are assigned to a therapy- and a control group, the kind of reading disorder the subjects have, must be known. This needs to be investigated in a pseudoword experiment (which is a statistical pretest) before the subjects are assigned to a therapy- and a control group. Only then is it possible to assign the subjects in such a way that in the therapy- and in the control group there are about the same number of subjects with the same type of reading disorders. Only if these methodological requirements are met, along with an adequate statistical approach, and the repeatability of the results has been demonstrated, can an experimental result be considered reliable.
